# Altered Functional Connectivity in the Reward Circuit in Betel Quid‐Dependent Chewers

**DOI:** 10.1111/adb.70180

**Published:** 2026-08-05

**Authors:** Yuegui Cao, Sisi Chen, Jiayu Li, Lingyu Kong, Jincheng He, Fulai Yuan, Jing He, Xueling Zhu

**Affiliations:** ^1^ Department of Radiology Xiangya Hospital, Central South University Changsha China; ^2^ Health Management Center Xiangya Hospital, Central South University Changsha China; ^3^ Department of Orthopaedics Xiangya Hospital, Central South University Changsha China; ^4^ National Clinical Research Center for Geriatric Disease Xiangya Hospital, Central South University Changsha China; ^5^ Hunan Singhand Intelligent Data Technology Co. Ltd Changsha China

**Keywords:** betel quid dependence, functional connectivity, resting‐state fMRI, reward circuit

## Abstract

Betel quid (BQ) is classified as a Group 1 carcinogen, underscoring a significant public health challenge. Neuroimaging evidence demonstrated that betel quid dependence (BQD) was linked to alterations in reward‐related brain regions. However, resting‐state functional connectivity (FC) in the reward circuit and their neural correlates with pathophysiological characteristics of BQD remain largely unexplored. We recruited 53 male BQD chewers and 53 male healthy controls (HCs) to participate in this study. Eleven reward circuit seeds, such as ventral striatum (VS), ventromedial prefrontal cortex (VMPFC), anterior cingulate cortex (ACC), posterior cingulate cortex (PCC) and others, were selected. Resting‐state functional magnetic resonance imaging (rs‐fMRI) data were analysed to evaluate FC between predefined seed regions and the whole brain. Association between aberrant FC and clinical features was further investigated. Relative to HCs, individuals with BQD exhibited altered FC in the reward circuit, mainly in VS, VMPFC, ACC, PCC and thalamus (Th). Correlation analysis indicated that FC between right Th and PCC was negatively correlated with BQD scales and dosage of BQ use in BQD subjects. Our findings provided the first empirical evidence that individuals with BQD showed aberrant resting‐state FC in the reward circuit. Additionally, decreased connectivity between right Th and PCC was negatively associated with dependence degree and dosage of BQ use. These results highlighted the importance of the reward circuit in the pathophysiology of BQD and suggested that reward circuit dysfunction could serve as a potential circuit‐level biomarker.

## Graphical abstract

Resting‐state fMRI identified abnormal functional connectivity within the reward circuit in individuals with betel quid dependence, involving the ventral striatum, prefrontal cortex, posterior cingulate cortex and thalamus. Reduced connectivity between the thalamus and posterior cingulate cortex was related to dependence severity and BQ dosage. These findings provide new insights into the neural mechanisms underlying BQD.

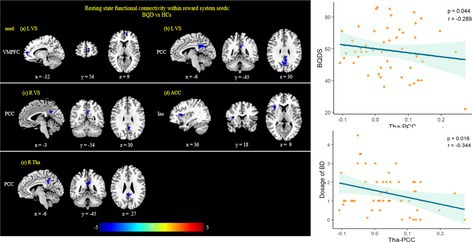

## 1 Introduction

Betel quid (BQ) is consumed by approximately 600 million people worldwide, ranking as the fourth most widely used self‐administered psychoactive substance, behind only caffeine, alcohol and nicotine [1, 2]. The usage of BQ is associated with serious health risks, which begin with localized oral damage such as tooth blackening, periodontitis [3] and potentially progressing to systemic diseases including cardiovascular and neurological disorders, as well as significantly elevating the risk of precancerous lesions and oral cancer [2, 4, 5]. According to the International Agency for Research on Cancer, BQ is classified as a Group 1 human carcinogen [6]. BQ mainly consists of areca nut, betel leaf and slaked lime [7]. As the key psychoactive substance in areca nut, arecoline acts as a competitive γ‐aminobutyric acid (GABA) inhibitor, a muscarinic acetylcholine receptor agonist and a monoamine oxidase‐A inhibitor, thereby disinhibiting and enhancing the activity of dopaminergic neurons within the mesolimbic reward pathway [8, 9, 10, 11]. Neuroimaging studies have revealed reward‐related function deficits in betel quid dependence (BQD) individuals [12]; however, the pathophysiological mechanism underlying the reward circuit is poorly understood.

Converging findings from animal models and human neuroimaging studies demonstrated that reward processing was mediated by a neural circuit centred on ventral striatum (VS), extending to ventral segmental area (VTA), ventromedial prefrontal cortex (VMPFC), posterior cingulate cortex (PCC), anterior cingulate cortex (ACC), anterior insula (Ins) and thalamus (Th) [13, 14, 15]. This circuit has been validated by a quantitative meta‐analysis conducted by Bartra et al. [16], which confirmed the existence of this core reward‐related circuit. The reward circuit originates from dopaminergic projections of VTA and primarily projects to nucleus accumbens (NAc) of VS [17] Striatal signals are transmitted via thalamus to medial cortex regions, including VMPFC, ACC and PCC, which in turn project back to basal ganglia [18]. Considering that the reward circuit has been identified as a crucial determinant for addiction [19], investigating its impairments in BQD is of great importance.

A growing body of multimodal neuroimaging studies identified significant alterations in brain regions associated with the reward circuit in BQD chewers. For instance, a voxel‐based morphometric analysis of structural magnetic resonance imaging data demonstrated reduced grey matter volume in bilateral VMPFC and Ins [20]. Using amplitude of low‐frequency fluctuation and regional homogeneity analyses, the study by Liu et al. revealed reduced spontaneous brain activity in ACC and MPFC among individuals with BQD [21]. In addition, diffusion tensor imaging analyses indicated compromised white matter integrity, specifically revealing that BQD chewers exhibited lower fractional anisotropy and higher mean diffusivity in Tha [22]. When [18]. F‐2‐fluoro‐2‐deoxy‐D‐glucose‐positron emission tomography was employed to explore metabolic pattern in brain regions of BQD chewers, one study reported reduced metabolism in MPFC and orbitofrontal cortex, along with increased metabolism in Tha [23]. To date, few studies employed functional connectivity (FC) analysis to elucidate the neurophysiological mechanisms underlying the reward circuit in BQD individuals.

Over the past few decades, resting‐state FC has been widely used to evaluate network‐level connectivity alternation in addiction research, and it has been consistently reported that individuals with addictive behaviours exhibit abnormal connectivity within the reward circuit [17]. Using seed‐based connectivity analysis, Zhang et al. identified decreased FC between VTA and NAc in subjects with internet gaming disorder [24]. In another chronic heroin addiction study, Ma and his colleagues observed increased FC between NAc and ACC but decreased connectivity between prefrontal cortex and ACC [25]. Furthermore, cigarette smokers demonstrated stronger Tha and ACC connectivity relative to healthy controls (HCs), and this enhanced connectivity was further linked to a higher urge to smoke [26]. These alterations in FC, especially within key reward‐related regions, highlighted that substance and behavioural addictions were characterized by altered FC in the reward circuit.

Emerging evidence suggested that BQD chewers displayed abnormal activity patterns of several large‐scale brain networks. One recent study investigated resting‐state functional and effective connectivity in the executive control network, which reported that BQD individuals showed disrupted intranetwork connectivity [27]. Another paper that directly examined FC of key nodes within the salience network revealed reduced FC of the salience network in BQD individuals when compared with HCs [28]. In addition, Huang et al. employed independent component analysis to compare global functional brain networks before and after BQ chewing, revealing that acute BQ use modulated FC of the frontal and default mode networks [29]. A systematic review investigating the mechanism of BQD exhibited that the reward circuit was the first affected in BQD individuals during the development of dependence [30]. However, no study directly examined BQD‐related resting‐state activity changes in the reward circuit in BQD chewers.

Therefore, the first aim of this study was to utilize resting‐state functional magnetic resonance imaging (rs‐fMRI) data to explore FC disruptions in the reward circuit among individuals with BQD when compared to HCs. The second aim was to assess the relationship between resting‐state reward circuit connectivity and BQD‐related psychological characteristics. We hypothesized that the BQD group would exhibit abnormal FC in the reward circuit, which would be correlated with clinical features.

## 2 Methods

### 2.1 Participants

The research protocol was approved by the Institutional Review Board of Xiangya Hospital, Central South University, Hunan, China. Prior to enrollment, all participants were informed about study procedures and provided their written informed consent. A total of 106 male participants were recruited to take part in this study. The baseline assessment collected data on age, years of education and other demographic characteristics. For participants with BQD, detailed information on their chewing behaviours was recorded, including duration of BQ use, age of first use and daily dosage. Information regarding the type of BQ consumed was also collected. All BQD participants primarily used commercially packaged BQ products commonly available in Hunan Province, China, which consisted mainly of areca nut and flavouring additives, with no tobacco, lime or other additives.

The diagnosis of BQD was based on the Diagnostic and Statistical Manual of Mental Disorders, Fifth Edition (DSM‐V) criteria [31]. Moreover, the severity of BQD was evaluated using the betel quid dependence scale (BQDS) [32]. Alcohol and nicotine addiction among participants were assessed using the Alcohol Use Disorders Identification Test and the Fagerström Test for Nicotine Dependence. In addition, all participants underwent a psychiatric evaluation conducted by experienced psychiatrists, and psychiatric disorders were assessed according to the diagnostic criteria of the Diagnostic and Statistical Manual of Mental Disorders, Fifth Edition.

**FIGURE 1:**
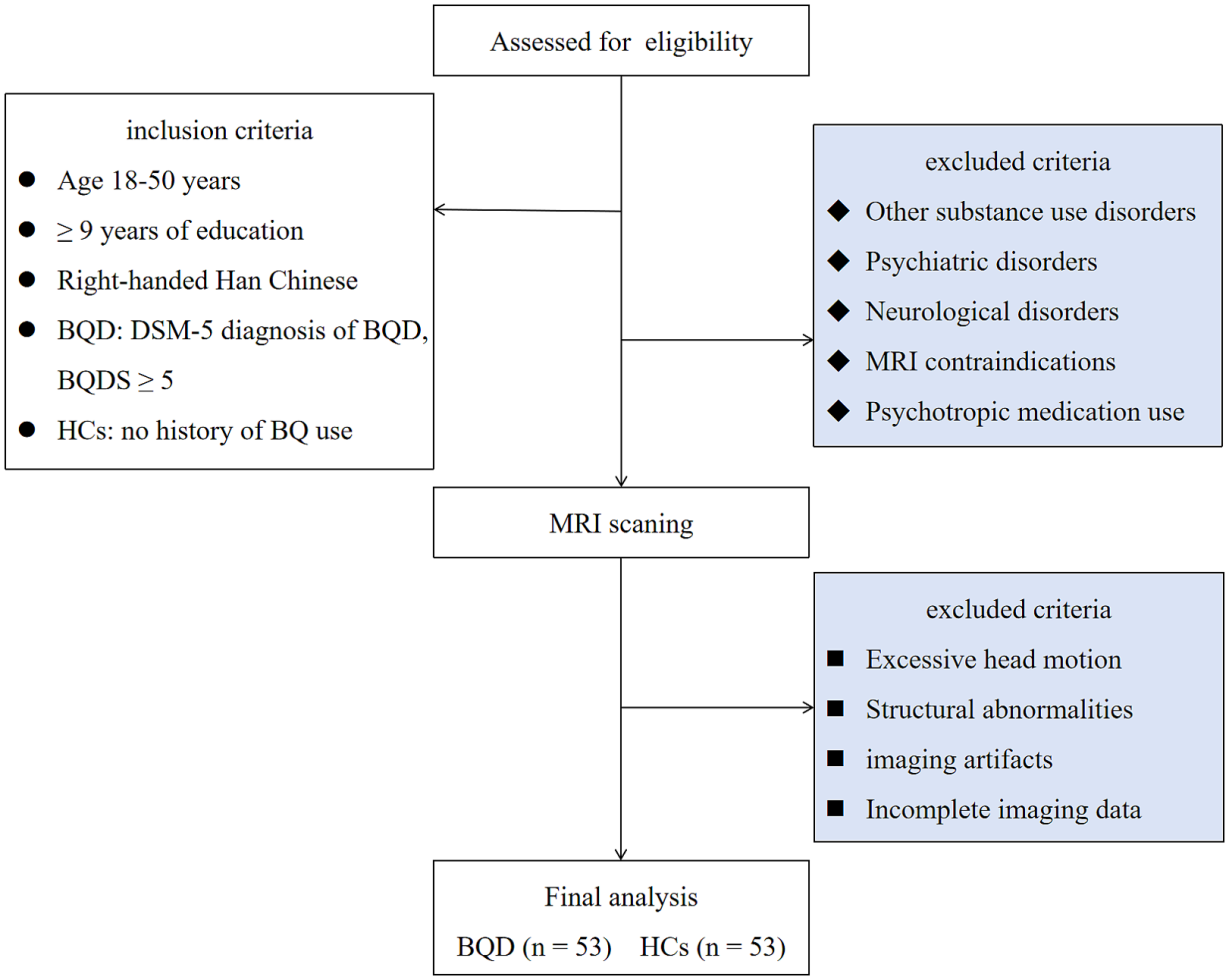
Participant recruitment and exclusion flowchart.

A cohort of 53 BQD chewers was recruited from the outpatient department of Xiangya Hospital, Central South University. The inclusion criteria comprised the following: (1) age between 18 and 50 years, (2) a minimum of 9 years of education, (3) right‐handedness, (4) Han Chinese ethnicity, (5) meeting the DSM‐V diagnostic criteria for BQD, (6) BQDS score ≥ 5 and (7) no history of BQ quit attempts in the past 6 months. Fifty‐three participants who had never used BQ were identified as HCs and were recruited from community in the Changsha City area. The inclusion criteria for the HCs were aligned with Criteria (1)–(4) as specified for the BQD group. The two groups shared the following exclusion criteria: (1) diagnosis of any substance use disorder other than BQD (e.g., alcohol use disorder, nicotine dependence or illicit drug use disorders); (2) a history of neurological disorders, including epilepsy, stroke, traumatic brain injury and brain tumours; (3) a lifetime diagnosis of any mental disorder, such as depressive disorders, anxiety disorders or personality disorders; (4) any contraindication to MRI scanning; and (5) current use of psychotropic medications (see Figure 1).

### 2.2 Image Acquisition

The acquisition of rs‐fMRI data was performed on a Siemens 3.0T Prisma scanner. All subjects were required to refrain from using any psychoactive substances for 24 h before examining. During scanning, participants were instructed to remain motionless, keep their eyes closed, and stay awake. To minimize scanner noise and reduce head motion, earplugs and foam padding were utilized. The resting‐state blood‐oxygen‐level‐dependent (BOLD) signals were acquired using a single‐shot gradient‐recalled echo‐planar imaging sequence. The imaging parameters were set as follows: repetition time (TR) = 2000 ms, echo time (TE) = 30 ms, flip angle (FA) = 66°, matrix size = 94 × 94, slice thickness = 2 mm, slice gap = 2 mm, number of slices = 75, 240 volumes, and scanning time = 480 s. Structural imaging data were acquired by magnetization‐prepared rapid gradient‐echo sequence, with the following parameters: TR = 2110 ms, TE = 3.18 ms, FA = 9°, inversion time (TI) = 1030 ms, matrix size = 320 × 320, slice thickness = 0.7 mm, slice gap = 0 mm and number of slices = 256.

### 2.3 Data Preprocessing

The processing steps for rs‐fMRI images were performed using the DPABI (Data Processing & Analysis for Brain Imaging, http://rfmri.org/dpabi) toolkit [33]. For each participant, the following processes were employed: (1) removal of the first 10 volumes to account for magnetic field stabilization and subject acclimation; (2) slice time correction and head motion realignment, with a scrubbing criterion of > 2 mm displacement and > 2° rotation; (3) spatial normalization to the Montreal Neurological Institute (MNI) template and resampling to a 3 × 3 × 3 mm^3^ isotropic voxel size; (4) spatial smoothing using a 6 mm full‐width‐at‐half‐maximum Gaussian kernel; (5) linear detrending to remove slow signal drifts; (6) regression of nuisance covariates, including the six motion parameters, cerebrospinal fluid, and white matter signals; and (7) temporal band‐pass filtering within 0.01–0.1 Hz.

### 2.4 Reward Circuit Seeds Selection

**FIGURE 2:**
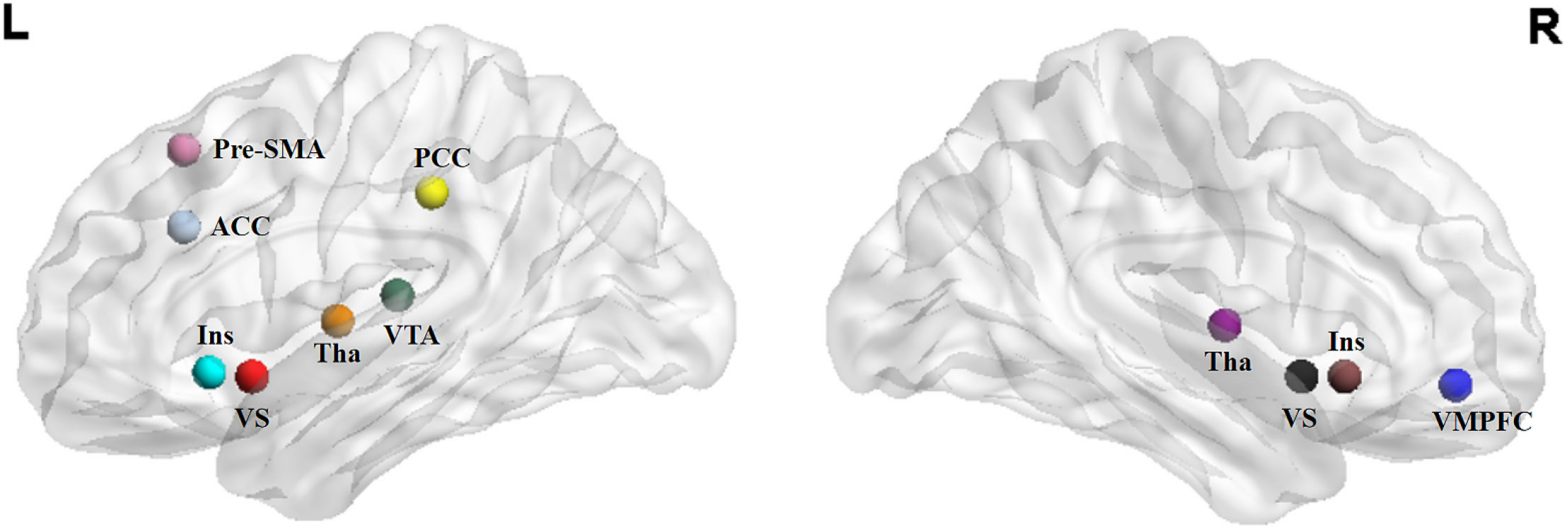
Seeds of the reward circuit in bilateral hemisphere. Abbreviations: ACC, anterior cingulate; Ins, anterior insula; L, left; PCC, posterior cingulate; Pre‐SMA, pre‐supplementary motor area; R, right; Th, thalamus; VMPFC, ventromedial prefrontal cortex; VS, ventral striatum; VTA, ventral segmental area.

A seed‐based voxel‐wise FC analysis was conducted with the reward circuit defined as regions of interest (ROIs). Eleven seed regions were derived from a meta‐analysis by Bartra et al. [16], which was widely adopted and validated in prior addiction research [34, 35, 36]. The coordinates of spherical ROIs in standardized MNI152 space were as follows: (1) left ventral striatum (VS; −12, 12, −7); (2) right ventral striatum (VS; 12, 10, −6); (3) ventromedial prefrontal cortex (VMPFC; 2, 46, −8); (4) left anterior insula (Ins; −30, 22, −6); (5) right anterior insula (Ins; 32, 20, −6); (6) posterior cingulate (PCC; −4, −30, 36); (7) ventral segmental area (VTA; −2, −22, 12); (8) anterior cingulate (ACC; −2, 28, 28); (9) pre‐supplementary motor area (Pre‐SMA; −2, 28, 46); (10) left thalamus (Th; −6, −8, 6); and (11) right thalamus (Th; 6, −8, 6). The radius of each seed was 5 mm (Figure 2).

### 2.5 FC Analysis

Resting‐state FC analyses were performed using predefined seed regions in REST (rs‐fMRI Data Analysis Toolkit, http://www.restfmri.net) [37]. The averaged BOLD time series were extracted, and Pearson correlation coefficients between each seed and voxels of the whole brain were computed. To improve normality for subsequent group‐level analysis, correlation coefficient maps were converted into *z*‐score maps using Fishers *r*‐to‐*z* transformation. We employed two‐sample *t*‐tests on *z*‐maps to investigate differences in FC between BQD and HCs groups. In the calculations, age and years of education were used as covariates to account for potential influence. Multiple comparison correction was applied using the Gaussian random field (GRF) theory (voxel‐wise *p* < 0.001, cluster‐level *p* < 0.05, two‐tailed).

### 2.6 Statistical Analysis

All analyses of demographic and clinical data were conducted using SPSS (version 24.0; SPSS Inc., Chicago, IL) to examine group differences between BQD and HCs participants. Prior to statistical analyses, normality and homogeneity of variance were assessed using the Shapiro–Wilk test and Levene's test, respectively. The two‐sample *t*‐tests were applied to compare continuous variables, such as age and years of education. Between‐group differences in FC were examined using two‐sample *t*‐tests, with age and years of education as nuisance covariates.

### 2.7 Correlation Analysis

Correlation analyses were conducted in SPSS to clarify the clinical meaning of findings. The mean values of clusters with aberrant FC were computed in the BQD group. Then we conducted partial correlation analyses to investigate relationships between *z*‐values of abnormal FC and clinical features, such as BQDS, duration of BQ, age of first BQ use and dosage of BQ. Age and years of education were added as nuisance covariates. The threshold for statistical significance was set at *p* < 0.05.

## 3 Results

### 3.1 Demographic and Clinical Data

Table 1 presented demographic and clinical characteristics of BQD and HCs. The two groups showed significant difference in terms of age (*t* = 5.96, *p* < 0.001) and years of education (*t* = −8.15, *p* < 0.001). To control for potential confounding impact of demographic difference, age and years of education were included as covariates in statistical analyses. For BQD group, the average BQDS score was 59.76, the average duration of BQ using was 16.09 years, the average age for onset of BQ chewing was 17.56 years and the average daily dosage of BQ consumption was 42.37 g.

**TABLE 1:** Demographic and clinical characteristics of BQD chewers and HCs (M ± SD).

Characteristics	BQD (*n* = 53)	HCs (*n* = 53)	*t*/*x* ^2^	*p*
Gender (male/female)	53/0	53/0		
Age (years)	34.65 ± 7.21	27.26 ± 6.78	5.96	< 0.001^a^
Education (years)	12.15 ± 2.39	15.96 ± 2.42	−8.15	< 0.001^a^
BQDS	59.76 ± 14.48	N/A		
Duration of BD use (years)	16.09 ± 6.83	N/A		
Age of first BD use (years)	17.56 ± 6.52	N/A		
Dosage of BD use (g/day)	42.37 ± 32.64	N/A		
AUDIT	3.47 ± 2.63	2.94 ± 2.15	1.14	0.41^a^
FTND	1.89 ± 1.72	1.36 ± 1.21	1.83	0.07^a^

Abbreviations: AUDIT, Alcohol Use Disorders Identification Test; BD, betel dependence; BQD, betel quid dependence; BQDS, Betel Quid Dependence Scale; FTND, Fagerström Test for Nicotine Dependence; HCs, healthy controls; M, mean; N/A, not applicable; SD, standard deviation.

^a^ The *p*‐value was obtained by a two‐sample *t*‐test.

### 3.2 FC Results

**FIGURE 3:**
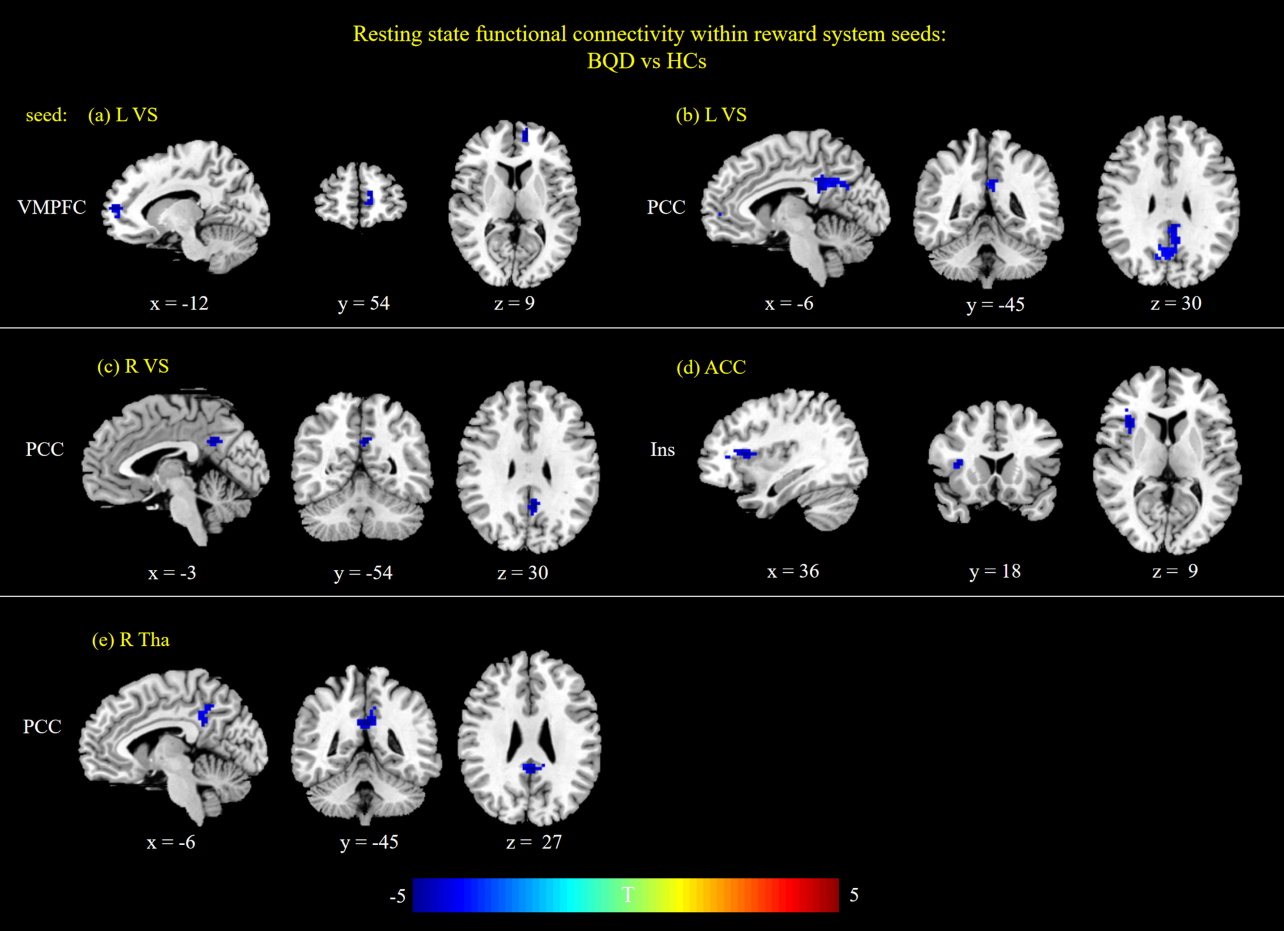
Resting‐state functional connectivity within the reward circuit in BQD chewers compared to HCs. BQD subjects showed significantly decreased functional connectivity in VS, VMPFC, PCC, ACC, Ins, and Tha (labelled blue) (GRF corrected, voxel *p* < 0.001, cluster *p* < 0.05). *T*‐value bar was presented below.

Two‐sample *t*‐tests (*p* < 0.05 with GRF correction) revealed significant intergroup differences in the reward circuit between BQD chewers and HCs. Relative to HCs, BQD subjects showed widely decreased FC in the reward circuit, including between left VS and left VMPFC, between left VS and left PCC, between right VS and left PCC, between left ACC and right Ins and between right Th and left PCC (see Table 2 and Figure 3). The remaining seeds demonstrated no significant FC with any whole brain areas.

**TABLE 2:** Brain regions with significantly differences in functional connectivity within the reward circuit between BQD chewers and HCs.

			Peak MNI coordinates			
Seeds	Brain region	Cluster size	*x*	*y*	*z*	*T*
L VS	L VMPFC	37	−12	60	9	−4.00
L VS	L PCC	107	−6	−39	24	−4.39
R VS	L PCC	32	−3	−54	30	−3.96
ACC	R Ins	41	36	18	9	−4.19
R Th	L PCC	62	−3	−45	27	−4.32

*Note:* Statistical significance was defined using Gaussian random field theory correction, with thresholds set at a voxel‐level of *p* < 0.001 and a cluster‐level of *p* < 0.05 (two‐tailed).

Abbreviations: ACC, anterior cingulate cortex; Ins, insula; L, left; MNI, Montreal Neurological Institute; PCC, posterior cingulate cortex; R, right; SMA, supplementary motor area; Th, thalamus; VMPFC, ventromedial prefrontal cortex; VS, ventral striatum.

### 3.3 Correlation Analysis Results

**FIGURE 4:**
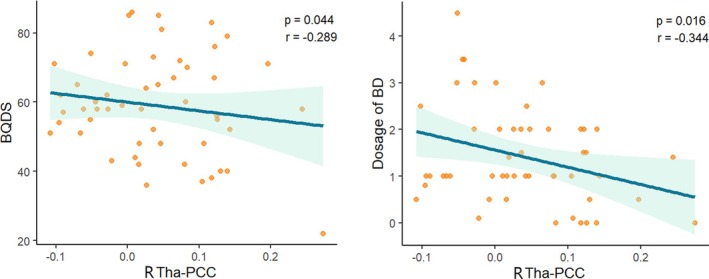
Partial correlation analysis results between functional connectivity in right Tha‐PCC and clinical characteristics in BQD individuals. The functional connectivity of right Tha and PCC was significantly negatively correlated with BQD scales (A). There was a significant negative correlation between functional connectivity in right Tha and PCC and dosage of BQ (B).

The partial correlation analysis result was shown in Figure 4. With age and years of education being controlled as covariants, the FC of right Th and PCC was negatively correlated with BQD score (*r* = −0.289, *p* = 0.044) and dosage of BQ use (*r* = −0.344, *p* = 0.016) in BQD chewers. No significant association was observed between other abnormal FC and clinical variables.

## 4 Discussion

In this study, we used seed‐based FC methodology to demonstrate a pattern of the reward circuit functional deficits in the BQD group, which aligned with our hypotheses. Compared to HCs, BQD subjects showed widespread abnormal FC in several reward pathways, especially in VS, VMPFC, ACC, PCC, Ins and Th. Furthermore, the resting‐state FC between the right Th and PCC was negatively correlated with BQD score and dosage of BQ use in BQD individuals. Our study provided the first evidence for altered resting‐state reward circuit activity in BQD individuals and revealed an association between BQ features and aberrant neural activity in the reward circuit.

A key finding of the present study was decreased FC between VS and VMPFC in BQD chewers. These two regions are typically recognized as central nodes in the reward circuit, supporting reward evaluation, subjective value and the development of habitual behaviour [38]. Anatomically and functionally, VS shares dense reciprocal connections with VMPFC, forming a critical substrate for processing drug rewards and reinforcing addictive behaviours [39, 40]. In fact, there has been a convergence of findings demonstrating functional abnormalities across these two brain regions in addictive behaviours. Neurobiological study indicated that exposure to addictive substances altered MPFC function and disrupted dopaminergic signalling in the VS, contributing to maladaptive reward processing and compulsive drug‐seeking behaviours [41]. Furthermore, prolonged substance use was associated with dysregulated dopamine dynamics in the VS, leading to altered reward sensitivity and increased vulnerability to relapse [42]. Our study suggested that dysregulation of VS and VMPFC played a pivotal role in the pathophysiology of BQD and expanded existing literature by identifying abnormal neural activity between VS and VMPFC in BQD chewers.

Moreover, the VS also showed decreased FC with PCC in BQD subjects, which may reflect impaired integration of subcortical reward signals into self‐referential processing and internal motivational context. In a cue‐exposure fMRI task study, individuals with gambling disorder or cocaine use disorder exhibited reduced connectivity in PCC in response to gambling and drug cues [43]. This alteration suggested a shift toward habit‐driven behaviour rather than self‐directed regulation [44]. Another study by Ma et al. indicated diminished FC between MPFC and PCC in heroin dependence individuals, which was correlated with the duration of heroin use [45]. Our observation of reduced VS‐VMPFC and VS‐PCC connectivity demonstrated a pattern of VS‐centred dysfunction within the reward circuit, reflecting impaired network‐level integration of reward‐related processing. Importantly, this VS‐centred network FC dysfunction provided novel neurobiological evidence for neural mechanisms underlying BQD.

We also observed that BQD subjects exhibited reduced FC between ACC and Insula. This hypoconnectivity may indicate a weakened top–down modulatory influence of motivational salience and interoceptive reward signals, potentially impairing functions related to addiction such as cognitive inhibition and decision‐making [46]. Neuroimaging studies repeatedly showed that individuals with substance use disorders exhibited altered FC in ACC [47]. For example, Liu et al. reported increased connectivity between ACC and reward network and decreased connectivity between ACC and default mode network in BQD chewers [48]. Additionally, Li and colleagues revealed that young male adult smokers showed decreased FC between ACC and Insula, and this was correlated with pack‐years of smoking [49]. Our findings revealed aberrant FC between ACC and insula, which further highlighted the involvement of these regions in the neural pathophysiology of BQD.

Furthermore, partial correlation analyses showed that attenuated FC between right Th and PCC was negatively correlated with BQD score and dosage of BQ use in BQD individuals, implicating that Th connectivity alteration may serve as a neural biomarker for substance dependence. Acting as a central relay station of the reward circuit, the Th underpins key components of addiction, including reward processing and motivated behaviour [50]. Previous research demonstrated that Th resting‐state FC was correlated with dependence severity and craving in nicotine use disorder [26]. Similarly, another investigation found that cue‐induced craving enhanced functional coupling of Th in cocaine use disorder, and this neural change was correlated with cocaine dependence severity [51]. Taken together, these findings confirmed our observation that aberrant Th connectivity was linked to core behavioural indices of addiction and highlighted that alterations in Th connectivity constituted a common neural feature of substance dependence.

Several limitations of this study need to be acknowledged. Firstly, the relatively small sample size and the inclusion of only male participants may restrict the generalizability of present findings, particularly to female populations. Future investigations should include larger samples with both sexes. Secondly, since participants were recruited from the same geographical region, this may constrain the extension of our conclusions to BQD populations from different cultural, ethnic, or geographical backgrounds. Future studies with more diverse and multicentre samples are needed to validate the reproducibility and broader applicability of the observed findings. Thirdly, although no significant differences were observed between groups, the potential influence of alcohol and tobacco consumption on brain connectivity measures cannot be entirely ruled out. In the future, more rigorous control of alcohol and tobacco use, or the inclusion of these variables as covariates in statistical analyses, may help clarify their potential confounding effects.

## 5 Conclusion

In conclusion, using seed‐based FC, we found hypoconnectivity (including VS, VMPFC, ACC, PCC, Ins, and Th) in BQD chewers when compared with HCs. Additionally, we also identified that the BQD score and dosage of BQ use were related to FC alternation between right Th and PCC in BQD subjects. Our results contributed to a deeper understanding of the reward circuit dysconnectivity and provided new insights into the neuropathological mechanisms underlying BQD.

## Author Contributions

Fulai Yuan conceptualized the research framework and acquired funding. Jing He was responsible for participant recruitment and data acquisition. Xueling Zhu designed the study, acquired funding and reviewed the manuscript. Yuegui Cao was involved in experimental implementation, data analysis and manuscript drafting. Sisi Chen provided assistance with experimental procedures. Lingyu Kong conducted literature searches. Jincheng He oversaw data management. All authors read the manuscript and approved the final version.

## Funding

This research received financial support from the National Natural Science Foundation of China (grant numbers: 62573429, 62372470, 62172440, 62473380, 62272481 and the The Science and Technology Innovation Program of Hunan Province (grant number: 2023RC1029).

## Conflicts of Interest

The authors declare no conflicts of interest.

## Data Availability

The datasets supporting the findings of this study are available from the corresponding author upon reasonable request.
